# Clinical History and Detectable Troponin Concentrations below the 99th Percentile for Risk Stratification of Patients with Chest Pain and First Normal Troponin

**DOI:** 10.3390/jcm10081784

**Published:** 2021-04-20

**Authors:** Agustín Fernández-Cisnal, Ernesto Valero, Sergio García-Blas, Vicente Pernias, Adela Pozo, Arturo Carratalá, Jessika González, José Noceda, Gema Miñana, Julio Núñez, Juan Sanchis

**Affiliations:** 1 Cardiology Department, University Clinic Hospital of València, Instituto de Investigación Sanitaria (INCLIVA), University of València, Centro de Investigación Biomédica en Red Enfermedades Cardiovaculares (CIBERCV), 46010 València, Spain; fecia82@gmail.com (A.F.-C.); ernestovaleropicher@hotmail.com (E.V.); sergiogarciablas@gmail.com (S.G.-B.); sainrep@hotmail.com (V.P.); adelapozogiraldez@gmail.com (A.P.); jessikabeg89@gmail.com (J.G.); gemineta@gmail.com (G.M.); yulnunez@gmail.com (J.N.); 2 Clinical Biochemistry Department, University Clinic Hospital of València, Instituto de Investigación Sanitaria (INCLIVA), 46010 València, Spain; carratala_art@gva.es; 3 Emergency Department, University Clinic Hospital of València, Instituto de Investigación Sanitaria (INCLIVA), 46010 València, Spain; noceda_jos@gva.es

**Keywords:** troponin, chest pain, acute coronary syndrome, clinical evaluation, ischemic heart disease

## Abstract

Decision-making is challenging in patients with chest pain and normal high-sensitivity cardiac troponin T (hs-cTnT; <99th percentile; <14 ng/L) at hospital arrival. Most of these patients might be discharged early. We investigated clinical data and hs-cTnT concentrations for risk stratification. This is a retrospective study including 4476 consecutive patients presenting to the emergency department with chest pain and first normal hs-cTnT. The primary endpoint was one-year death or acute myocardial infarction, and the secondary endpoint added urgent revascularization. The number of primary and secondary endpoints was 173 (3.9%) and 252 (5.6%). Mean hs-cTnT concentrations were 6.9 ± 2.5 ng/L. Undetectable (<5 ng/L) hs-cTnT (*n* = 1847, 41%) had optimal negative predictive value (99.1%) but suboptimal sensitivity (90.2%) and discrimination accuracy (AUC = 0.664) for the primary endpoint. Multivariable analysis was used to identify the predictive clinical variables. The clinical model showed good discrimination accuracy (AUC = 0.810). The addition of undetectable hs-cTnT (≥ or <5 ng/L; HR, hazard ratio = 3.80; 95% CI, confidence interval 2.27–6.35; *p* = 0.00001) outperformed the clinical model alone (AUC = 0.836, *p* = 0.002 compared to the clinical model). Measurable hs-cTnT concentrations (between detection limit and 99th percentile; per 0.1 ng/L, HR = 1.13; CI 1.06–1.20; *p* = 0.0001) provided further predictive information (AUC = 0.844; *p* = 0.05 compared to the clinical plus undetectable hs-cTnT model). The results were reproducible for the secondary endpoint and 30-day events. Clinical assessment, undetectable hs-cTnT and measurable hs-cTnT concentrations must be considered for decision-making after a single negative hs-cTnT result in patients presenting to the emergency department with acute chest pain.

## 1. Introduction

Chest pain is a frequent cause of emergency department visits. However, only a minority of these patients is diagnosed with acute myocardial infarction (AMI) or experiences cardiac events [1]. The advent of high-sensitivity cardiac troponin (hs-cTn) assays has improved diagnostic accuracy. Hs-cTn elevation implies a high risk requiring a complete diagnostic work-up and close monitoring regardless of a final diagnosis of AMI or non-ischemic myocardial injury [2]. The scenario is perhaps more challenging when hs-cTn concentrations are normal (below the 99th percentile) at hospital arrival. Overall, these are lower-risk patients, but their event rate is far from negligible [3,4]. 

Several algorithms using hs-cTn have been developed, such as the undetectable hs-cTn, 0/1-h, and 0/2-h algorithms [5,6,7,8]. In all these tools, decision-making is centered on hs-cTn concentrations. The performance of hs-cTn algorithms is supported by substantial evidence [9], yet there is still room for improvement [10]. Hs-cTn algorithms create rule-in or rule-out thresholds, but individualized decisions also require clinical judgment. The valuable contribution of troponin as a biomarker should not overshadow careful clinical assessment. Indeed, clinical scores might be a valuable complement to hs-cTn tools [11,12,13].

Unlike most other research in the field, this study focused on patients presenting at the emergency department with acute chest pain without signs of ischemia in the ECG (electrocardiogram) and having normal initial hs-cTnT concentrations (below the 99th percentile). Conceivably, most of these patients might be discharged early from the emergency department without further evaluation. We aimed to evaluate the potential role of clinical data, undetectable hs-cTnT (below the detection limit), and measurable hs-cTnT concentrations (above the detection limit but below the 99th percentile), for one-year outcomes.

## 2. Materials and Methods

### 2.1. Study Design

This retrospective study involved consecutive adult patients presenting at the emergency department of the University Clinic Hospital of València (Spain) with a chief complaint of chest pain, without persistent ST-segment elevation in the initial ECG and with normal first hs-cTnT concentrations (<99th percentile = 14 ng/L; Roche Diagnostics, Basel, Switzerland). The study period was from July 2016 to February 2019. Exclusion criteria were ECG evidence of ischemia defined by transient ST-segment deviation, potential causes of myocardial increased oxygen demand such as tachyarrhythmias (>100 beats/min) or bradyarrhythmias (<50 beats/min), and non-ischemic causes of chest pain after diagnostic work-up undertook in the emergency department, such as non-ischemic structural heart disease, pericardial disease, aortic dissection, or extracardiac disease. Data were searched in the electronic medical records of emergency department visits using the following methodology: first, identifying all patients during the study period over 18 years old with hs-cTnT determination at hospital arrival and having a normal result. Second, selecting patients with chest pain as a chief complaint without persistent ST-segment elevation. Third, applying the exclusion criteria. Of 6104 patients screened, 4476 were eligible for the study (Figure S1). A preliminary analysis of the performance of GRACE (Global Registry of Acute Coronary Events ), TIMI (Thrombolysis in Myocardial Infarction), and HEART (History, EKG, Age, Risk factors, and Troponin) scores in the first 2254 patients was previously published [13]. The study was reviewed and approved by the Clinical Research Ethics Committee of the University Clinic Hospital of València.

Patients were managed according to emergency department practice. Therefore, additional hs-cTnT measurements, non-invasive ischemia tests, and admission or discharge decisions were at the attending physician’s discretion. The following variables were collected at admission: chest pain characteristics (effort-related chest pain at admission or during the previous week, recurrence within 24 h), coronary risk factors, prior history of ischemic heart disease (prior myocardial infarction, coronary revascularization, or admission for heart failure), extracardiac atherosclerotic disease (peripheral artery disease, previous stroke), systolic blood pressure and heart rate, abnormal ECG (persistent ST-segment depression < 0.5 mm, T wave inversion > 1, left bundle branch block, permanent pacemaker, or atrial fibrillation), time from chest pain onset to first blood sample, and routine blood tests (hs-cTnT, creatinine, and hemoglobin).

### 2.2. Hs-cTnT Assay

The analytic performance of the hs-cTnT assay was as follows: The limit of blank was 3 ng/L, the limit of detection was 5 ng/L, and the limit of quantification (the lowest analyte concentration that can be reproducibly measured with a coefficient of variation of 10% or less) was 13 ng/L. The limit of blank and limit of detection was determined according to the CLSI (Clinical and Laboratory Standards Institute) EP17-A requirements. The limit of quantitation was determined using the result of functional sensitivity testing. The 99th centile of a healthy reference population recommended as a positivity threshold for diagnosing acute myocardial infarction was 14 ng/L (ng/L). Samples were performed in the laboratory as soon as the blood collection was obtained. Plasma lithium heparin samples were used.

### 2.3. Endpoints

The primary endpoint was death or AMI at one-year follow-up. AMI was defined by a hs-cTnT rising and/or falling pattern with at least one value above the 99th percentile, along with clinical evidence of acute myocardial ischemia. The rising and/or falling pattern was considered as serial changes >50% when the initial hs-cTnT was below the 99th percentile and >20% when it was above [14]. Since the initial hs-cTnT was below the 99th percentile in all patients at the index episode, the >50% criterion was taken to diagnose index AMI. The secondary endpoint was defined by death, AMI, or urgent coronary revascularization at one year. Revascularization was defined as urgent if performed during hospitalization for chest pain suggestive of ischemic origin and indicated to avoid further deterioration. Endpoints were also analyzed at 30-day follow-up. We registered follow-up data from hospital records or outpatient departments, contacting the patient or the general physician if patients did not return to the hospital or outpatient department.

### 2.4. Statistical Analysis

Continuous variables were expressed by mean and standard deviation, or median with the interquartile interval, while categorical variables were expressed by absolute values and percentages. Hs-cTnT values were dichotomized according to the detection limit (≥5 ng/L, Roche Diagnostics, Basel, Switzerland). Sensitivity, specificity, and negative and positive predictive values of detectable hs-cTnT for the primary and secondary endpoints were estimated. Univariate and multivariable Cox regression analyses (backward conditional method, exit at *p* < 0.1) were carried out to identify, which clinical variables were related to the primary endpoint. Clinical variables shown in Table 1 were tested. The hazard ratio (HR) and 95% confidence intervals (CI) were calculated. Next, we calculated the area under the receiver operating characteristic curve (AUC), with the 95% CI (bootstrapping method with 1000 iterations) of 3 predictive models: (1) clinical model using only clinical data; (2) addition of undetectable hs-cTnT to the clinical model, introducing hs-cTnT as a dichotomized variable (≥ or <5 ng/L); and (3) addition of hs-cTnT as a continuous variable according to the measured concentration (above the detection limit but below the 99th percentile) assigning any troponin concentration below the detection limit the same value (4.9 ng/L). The AUCs were compared using the Delong test. Finally, we evaluated risk reclassification using the integrated discrimination improvement (IDI) and continuous net reclassification improvement (NRI) indexes.

Statistical analysis was performed using SPSS version 20.0 software (SPSS, Inc., Chicago, IL, USA), “pROC” and “survIDINRI” R packages [15,16].

## 3. Results

### 3.1. Patient Population, Management, and Follow-Up

Table 1 shows the characteristics of the patient population. The mean age was 56 ± 16 years, and 53% were male. Mean hs-cTnT values were 6.9 ± 2.5 ng/L. A second hs-cTnT determination was indicated in 1438 patients (32%). The total number of patients hospitalized at the index episode was 329 (7.4%). An invasive coronary angiogram was carried out in 83 (4.1%) patients.

Follow-up was one year. After discharge, 40 (0.9%) patients were lost to follow-up. The median follow-up in lost patients was 185 days (130–232 interquartile intervals). A total of 173 (3.9%) patients experienced the primary endpoint (42 deaths and 131 non-fatal acute myocardial infarctions) and 252 (5.6%) the secondary endpoint at one year. The 30-day event rate was 92 (2.1%) for the primary endpoint (89 of them at the index episode) and 155 (3.5%) for the secondary endpoint (151 of them at the index episode).

### 3.2. Undetectable Hs-cTnT Concentrations

A total of 1847 (41%) patients showed undetectable (<5 ng/L) hs-cTnT levels. Sensitivity, specificity, negative, and positive predictive values of hs-cTnT ≥ 5 ng/L for the endpoints are presented in Table 2. Overall, the negative predictive value was optimal (around 99% for all endpoints). Sensitivity, however, was lower at around 90% for both the primary and secondary endpoints, at one year and 30 days. As expected, positive predictive value and specificity were very low. Discrimination accuracy of undetectable hs-cTnT was modest (AUC 0.664; 95% CI: 0.640–0.687).

### 3.3. Clinical Data and hs-cTnT

**Figure 1:**
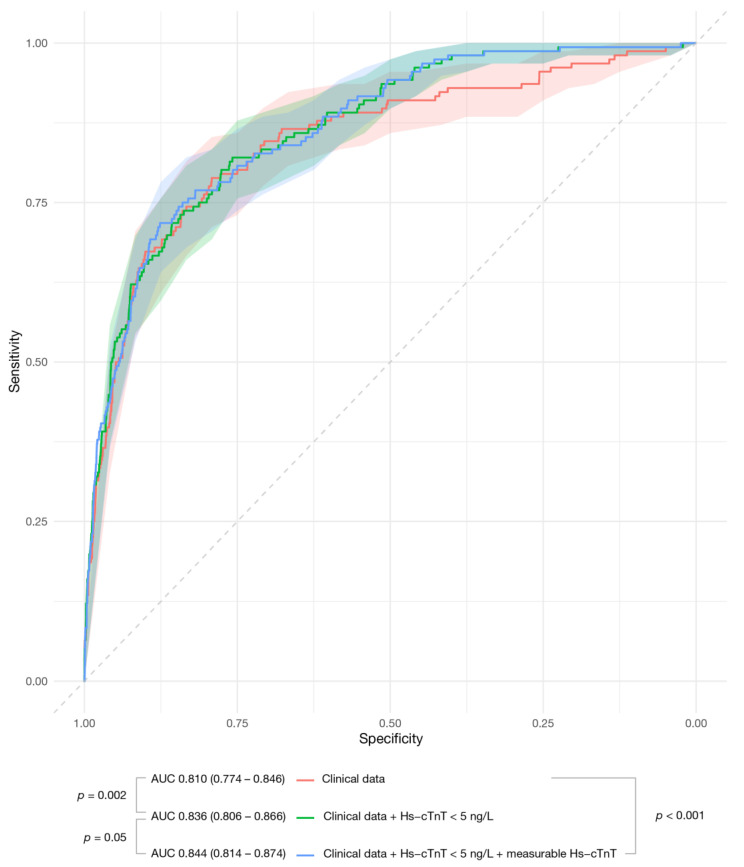
Discrimination accuracy (AUC with the 95% confidence intervals) of the predictive models for the primary endpoint. AUC = area under the receiver operating characteristic curve.

Table S1 presents the clinical variables independently associated with the primary endpoint: hypercholesterolemia, previous myocardial infarction, effort-related chest pain, chest pain recurrence within 24 h, admission systolic blood pressure, ST-segment depression, hemoglobin, and creatinine. The clinical model showed good discrimination accuracy for the primary endpoint (AUC = 0.810, 95% CI 0.775–0.846) (Table 3, Figure 1). However, undetectable hs-cTnT added significant predictive information (HR = 3.80; 95% CI 2.27–6.35; *p* = 0.00001) and improved model performance (AUC = 0.836, 95% CI 0.806–0.866; *p* = 0.002; in comparison with the clinical model alone). When introducing measurable hs-cTnT concentrations (above the detection limit but below the 99th percentile), undetectable hs-cTnT remained significant (HR = 2.36; 95% CI 1.06–4.20; *p* = 0.004), and measurable hs-cTnT gave additional information (per 0.1 ng/L; HR = 1.13; CI 1.06–1.20; *p* = 0.0001). The AUC showed the best discrimination accuracy (AUC = 0.844, 95% CI 0.814–0.874; *p* = 0.05 in comparison with the clinical plus undetectable hs-cTnT model). Overall, hs-cTnT concentrations alone were inferior to clinical data (AUC: 0.739, 95% CI 0.704–0.774; *p* = 0.002 in comparison with the clinical model); however, as expressed in Table 3 and Figure 1, hs-cTnT concentrations added meaningful information to clinical data. The results were quite similar for the secondary endpoint (Table 3). Here, the inclusion of measurable hs-cTnT also improved model performance (*p* = 0.04 compared to the clinical plus undetectable hs-cTnT model). In terms of 30-day events, measurable hs-cTnT did not provide significant information on top of clinical data and undetectable hs-cTnT (Table 3).

According to IDI and continuous NRI, the addition of undetectable hs-cTnT reclassified the risk over the clinical model (Table 4). Measurable hs-cTnT did not provide further risk reclassification.

### 3.4. Subgroup Analysis

Table 5 shows the results of the subgroup analysis according to age (≥70 or <70 years), gender, early or late presenters (≤180 or >180 min from pain onset to blood sample), and renal function (creatinine < or ≥1.3 mg/dL). The pattern was quite similar to that observed in the whole population. Measurable hs-cTnT outperformed undetectable hs-cTnT alone in late presenters. Remarkably, few patients (3.1%) had renal insufficiency, and hs-cTnT lacked a predictive value in this subgroup.

## 4. Discussion

The two most distinctive characteristics of this study were the focus on patients with chest pain and normal first hs-cTnT at hospital arrival, and follow-up extended to one year. The main findings were: (1) Undetectable hs-cTnT alone had suboptimal sensitivity and discrimination accuracy for one-year and 30-day outcomes. (2) However, undetectable hs-cTnT added meaningful information to clinical data for risk assessment. (3) The best predictive ability was achieved considering not only whether hs-cTnT is above or below the detection limit but also the measurable values of hs-cTnT between the detection limit and the 99th percentile. Therefore, early risk stratification in these patients should not merely be based on whether hs-cTnT concentrations were detectable or not. Both clinical evaluation and the amount of hs-cTnT below the 99th percentile must be considered. These data should be taken into account for decision-making after a single negative hs-cTnT result in patients presenting to the emergency department with acute chest pain.

### 4.1. Undetectable hs-cTnT

Decision-making in patients with chest and normal hs-cTnT at hospital arrival is challenging. Overall, this subset is considered a low-risk population. Our data show a 3.9% rate of death or myocardial infarction and a 5.6% rate of death, myocardial infarction, or urgent revascularization at one year. Identifying which patients warrant hospitalization and which can be discharged but under close surveillance in the outpatient cardiology unit for a final diagnosis is crucial. The undetectable hs-cTn algorithm (hospital discharge if hs-cTn below the detection limit) is a very attractive approach, mainly because of its rapidity and simplicity [5,17,18]. A meta-analysis showed excellent negative predictive value, but sensitivity results showed some heterogeneity among centers [5]. In a recent study, 2.2% of patients with ST-segment elevation AMI had hs-cTnT concentration below the detection limit at presentation [19]. We found optimal negative predictive value, around 99%, but lower sensitivity at about 90%, for not only one-year but also 30-day events. Given the low prevalence of events, sensitivity is as important as negative predictive value. These data do not deny the importance of undetectable hs-cTnT as an indicator of lower risk. However, adding measurable hs-cTnT values improved predictive ability over the undetectable hs-cTnT approach; in other words, although hs-cTnT concentrations were below the 99th percentile, the higher they were, the worse the prognosis. In other studies, patients with detectable hs-cTn below the 99th had a higher risk of death and AMI at one year in comparison with patients with undetectable hs-cTn [4,20,21]. Furthermore, in another scenario such as cardiovascular risk stratification in the general population, measurable detectable concentrations of troponin within the reference values added incremental risk prediction over well-established prognosticators and could also be a marker of myocardial remodeling [22,23].

### 4.2. Clinical Data

Clinical data are of paramount importance for decision-making in patients with acute chest pain [3]. Clinical scores, such as the TIMI and HEART scores, which stem from before the high-sensitivity troponin era, can contribute to a better risk stratification [13,24,25]. In contrast, the GRACE score seems to be of limited value in low-risk patients such as those with initial normal hs-cTnT [13]. An extended algorithm, including clinical and ECG data, complemented the hs-cTnT 0/1-h algorithm for patient triage [26]. Clinical variables found to have prognostic value in our population were the previous history of hypercholesterolemia or myocardial infarction, effort-related chest pain and recurrence within 24 h, higher systolic blood pressure at admission (probably as a surrogate for hypertension), ST-segment depression, hemoglobin, and creatinine. In the graphical abstract, we proposed a management algorithm: (1) High-risk patients according to clinical data should be cautiously evaluated. (2) Low-risk patients with undetectable hs-cTnT could be early discharged. (3) In low-risk patients with detectable hs-cTnT, the amount of measured hs-cTnT might be considered for decision making. Further studies combining detailed clinical data with hs-cTnT concentrations through machine learning could refine risk stratification [27].

### 4.3. Subgroup Analysis

The main results were reproduced across subgroups defined by age, gender, time from pain onset to blood sample, and creatinine levels. Measurable hs-cTnT contributed to risk stratification, especially in late presenters. As expected, few patients with renal insufficiency had normal troponin (3%). Remarkably, hs-cTnT lacked predictive value in these patients. Detectable troponin concentrations are often observed in renal dysfunction in the absence of ischemic heart disease, which might interfere with predictive accuracy [14,28].

### 4.4. Limitations

Although all consecutive patients during the study period were screened, the results of the study might be subject to some bias inherent to its retrospective design, as patient management was at the discretion of the attending physician. Besides, the study could be underpowered for 30-day events or subgroup analysis.

## 5. Conclusions

Patients presenting at the emergency department with acute chest pain and initial normal hs-cTnT concentrations have a non-negligible event rate at one year. Undetectable hs-cTnT alone showed suboptimal sensitivity for early risk stratification. However, combining clinical assessment, undetectable hs-cTnT, and measurable hs-cTnT concentrations below the 99th percentile (the higher they were, the worse the prognosis) could facilitate initial triaging of patients to further evaluation at the emergency department or early discharge with or without additional control at the outpatient cardiology unit.

## Figures and Tables

**Table 1:** Characteristics of the patient population (*n* = 4476).

**Reported Risk Factors**	
Age (years)	56 ± 16
Males	2380 (53%)
Current smokers	1142 (26%)
Hypertension	1812 (41%)
Hypercholesterolemia	1911 (43%)
Diabetes mellitus	603 (14%)
Family history of early ischemic heart disease	143 (3.2%)
**Reported Patient History**	
Previous myocardial infarction	523 (12%)
Previous percutaneous coronary intervention	448 (10%)
Previous coronary bypass surgery	59 (1.3%)
Previous admission for heart failure	111 (2.5%)
Peripheral artery disease	60 (1.3%)
Previous stroke	141 (3.2%)
**Chest Pain Characteristics at Presentation**	
Effort-related chest pain in the previous week	481 (11%)
Recurrent chest pain in the last 24 h	289 (6.5%)
**Physical Measures at Presentation**	
Admission systolic blood pressure (mmHg)	139 ± 21
Admission diastolic blood pressure (mmHg)	80 ± 14
Admission heart rate (beats/minute)	79 ± 15
**Electrocardiogram at Presentation**	
ST-segment depression ≥ 0.5 mm	155 (3.5%)
T-wave inversion ≥ 1 mm	296 (6.6%)
Admission atrial fibrillation (ventricular rate < 100 beats/min)	165 (3.7%)
Left bundle branch block	67 (1.5%)
Permanent pacemaker	27 (0.6%)
**Troponin**	
Admission hs-cTnT (ng/L)	6.9 ± 2.5
Admission undetectable hs-cTnT (<5 ng/L)	1847 (41%)
Time from chest pain onset to hs-cTnT determination (minutes) *	270 (162 to 655)
Second hs-cTnT determination	1438 (32%)
**Other Admission Blood Tests**	
Hemoglobin (g/dL)	14.1 ± 1.5
Creatinine (mg/dL)	0.85 ± 0.2
**Management at the Index Episode**	
Hospitalization at the index episode	329 (7.4%)
Exercise testing	122 (2.7%)
Stress cardiac magnetic resonance	98 (2.3%)
Invasive coronary angiogram	183 (4.1%)

Data are presented as *n* (%), or mean ± standard deviation, or median (lower-quartile to upper-quartile). * Missing values = 193. Hs-cTnT = high-sensitivity cardiac troponin T.

**Table 2:** Negative and positive predictive values (NPV and PPV), sensitivity (S), and specificity (Sp) of hs-cTnT at the detection limit (5 ng/L), for the primary (death or acute myocardial infarction) and secondary (death or acute myocardial infarction or urgent revascularization) endpoints.

One Year	NPV	PPV	S	Sp
Primary endpoint	99.1 (98.5 to 99.4)	5.9 (5.1 to 6.9)	90.2 (84.8 to 93.8)	42.5 (41.1 to 44.0)
Secondary endpoint	98.8 (98.2 to 99.2)	8.7 (7.7 to 9.9)	91.3 (87.1 to 94.2)	43.2 (41.7 to 44.7)
30 days				
Primary endpoint	99.5 (99.1 to 99.7)	3.2 (2.6 to 3.9	90.3 (82.6 to 94.8)	41.9 (40.5 to 43.4)
Secondary endpoint	99.3 (98.8 to 99.6)	5.4 (4.6 to 6.4)	91.7 (86.3 to 95.1)	42.5 (41.0 to 43.9)

**Table 3:** Discrimination accuracy (area under the receiver operating characteristic curve) of the predictive models for the primary (death or acute myocardial infarction) and secondary (death or acute myocardial infarction or urgent revascularization) endpoints.

One-Year	Clinical Data	Clinical Data + Hs-cTnT ≥ 5 ng/L	Clinical Data + Hs-cTnT ≥ 5 ng/L + Measured Hs-cTnT
Primary endpoint	0.810 (0.775–0.846)	0.836 (0.806–0.866) 0.002 ^a^	0.844 (0.814–0.874) 0.05 ^b^
Secondary endpoint	0.847 (0.820–0.875)	0.871 (0.849–0.896) <0.0001 ^a^	0.876 (0.854–0.898) 0.04 ^b^
30 days			
Primary endpoint	0.817 (0.767–0.867)	0.839 (0.798–0.880) 0.05 ^a^	0.840 (0.799–0.881) 0.6 ^b^
Secondary endpoint	0.847 (0.811–0.883)	0.866 (0.837–0.895) 0.05 ^a^	0.866 (0.837–0.896) 0.9 ^b^

^a^ In comparison with the clinical model. ^b^ In comparison with the clinical + Hs-cTnT ≥ 5 ng/L model.

**Table 4:** Risk reclassification for the primary endpoint after combining hs-cTnT and clinical data.

Adding Hs-cTnT ≥ 5 ng/L to Clinical Data		Adding Measured hs-cTnT to Clinical Data Plus Hs-cTnT ≥ 5 ng/L	
IDI	Continuos NRI	IDI	Continuos NRI
0.0090 (−0.0001–0.019) *p* = 0.05	0.2859 (0.1867–0.3422) *p* = 0.001	0.006 (−0.001–0.0211) *p* = 0.1	−0.0277 (−0.1106–0.0560) *p* = 0.6

IDI = integrated discrimination improvement. NRI = continuous net reclassification improvement.

**Table 5:** Discrimination accuracy (C-statistic) of the predictive models for the primary (death or acute myocardial infarction) endpoint in different subgroups.

	Clinical Data	Clinical Data + Hs-cTnT ≥ 5 ng/L	Clinical Data + Hs-cTnT ≥ 5 ng/L + Measured Hs-cTnT
Age			
<70 years (*n* = 3487)	0.808 (0.762–0.853)	0.841 (0.804–0.879) 0.008 ^a^	0.848 (0.811–0.885) 0.1 ^b^
≥70 years (*n* = 989)	0.788 (0.709–0.864)	0.794 (0.720–0.868) 0.003 ^a^	0.811 (0.741–0.880) 0.3 ^b^
Gender			
Female (*n* = 2096)	0.853 (0.803–0.903)	0.874 (0.831–0.917) 0.1989 ^a^	0.884 (0.843–0.925) 0.06 ^b^
Male (*n* = 2380)	0.800 (0.753–0.846)	0.819 (0.778–0.860) 0.02 ^a^	0.822 (0.780–0.864) 0.5 ^b^
Time from chest pain onset to blood sample ^c^			
≤180 min (*n* = 1461)	0.781 (0.728–0.834)	0.805 (0.760–0.851) 0.04 ^a^	0.806 (0.760–0.852) 1 ^b^
>180 min (*n* = 2822)	0.848 (0.798–0.897)	0.874 (0.834–0.913) 0.04 ^a^	0.889 (0.852–0.926) 0.03 ^b^
Admission creatinine			
<1.3 mg/dL (*n* = 4337)	0.800 (0.761–0.838)	0.828 (0.796–0.860) 0.002 ^a^	0.837 (0.805–0.869) 0.07 ^b^
≥1.3 mg/dL (*n* = 139)	0.924 (0.870–0.977)	0.928 (0.876–0.981) 0.6 ^a^	0.926 (0.872–0.980) 0.3 ^b^

^a^ In comparison with the clinical model. ^b^ In comparison with the clinical + Hs-cTnT ≥5 ng/L model. ^c^ 193 missing values not included.

## Data Availability

The data presented in this study are available on request from the corresponding author. The data are not publicly available due to privacy and ethical reasons.

## Supplementary Materials

The following are available online at https://www.mdpi.com/article/10.3390/jcm10081784/s1. Figure S1: Patient screening and eligibility, Table S1: Predictive clinical data for the primary endpoint (one-year death or myocardial infarction).
