# Supplementary material for: Clinical History and Detectable Troponin Concentrations below the 99th Percentile for Risk Stratification of Patients with Chest Pain and First Normal Troponin
Source: J Clin Med. 2021 Apr 20;10(8):1784. doi: 10.3390/jcm10081784 (PMC8073372; doi:10.3390/jcm10081784)

Table S1

Predictive clinical data for the primary endpoint (one-year death or myocardial infarction)

Variable	Hazard ratio	95% Confidence intervals	p
Hypercholesterolemia	1.715	1.210 to 2.430	0.002
Previous myocardial infarction	3.148	1.897 to 5.225	<0.001
Effort-related chest pain	1.778	1.238 to 2.552	0.002
Chest pain recurrence within 24 hours	4.895	3.451 to 6.943	<0.001
Systolic blood pressure (per 10 mmHg)	1.014	1.007 to 1.020	<0.001
ST-segment depression	3.113	2.039 to 4.754	<0.001
Creatinine (per 0.1 mg/dL)	2.462	1.345 to 4.508	0.004
Haemoglobin per (g/dL)	0.890	0.803 to 0.986	0.026

Supplemental Figure 1

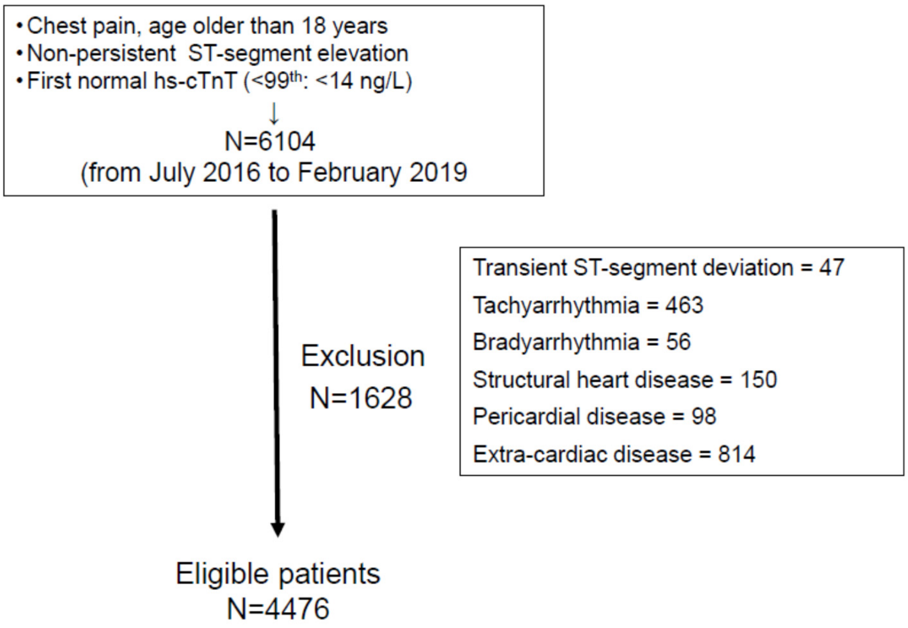

Supplement: Supplementary file 1 [file jcm-10-01784-s001.zip › jcm-1191827-supplementary.pdf]
